# A comprehensive in silico analysis of mutation spectrum of maple syrup urine disease (MSUD) genes in Iranian population

**DOI:** 10.22099/mbrc.2024.49847.1958

**Published:** 2024

**Authors:** Nahid Rezaie, Saeedeh Sadat Ghazanfari, Teymoor Khosravi, Fatemeh Vaghefi, Morteza Oladnabi

**Affiliations:** 1 Student Research Committee, Golestan University of Medical Sciences, Gorgan, Iran; 2 Mashhad University of Medical Sciences, Mashhad Branch, Islamic Azad University, Mashhad, Iran; 3 Gorgan Congenital Malformations Research Center, Golestan University of Medical Sciences, Gorgan, Iran; 4 Department of Medical Genetics, School of Advanced Technologies in Medicine, Golestan University of Medical Sciences, Gorgan, Iran; 5 Ischemic Disorders Research Center, Golestan University of Medical Sciences, Gorgan, Iran

**Keywords:** In silico analysis, Maple syrup urine disease, Mutations, Pathogenicity

## Abstract

Maple syrup urine disease (MSUD) represents an infrequent metabolic disease precipitated by an insufficiency of the enzymatic complex known as branched-chain alpha-keto acid dehydrogenase. MSUD can be classified as classic (severe), intermediate, or intermittent based on the severity of the condition. The disease is associated with mutations in several genes, including *BCKDHA*, *BCKDHB*, *DBT*, and *DLD*. This study aimed to investigate the genetic landscape of MSUD in Iranian patients and explore the clinical implications of identified gene variants. A comprehensive analysis was conducted using various molecular techniques and bioinformatics tools to predict protein stability, pathogenicity, amino acid conservation, and secondary/tertiary structure. The in silico analysis highlighted high-risk pathogenic variants and provided insights into their potential impact on protein structure and function. Furthermore, the predicted 3D structures of wild-type and mutant proteins elucidated structural differences. Protein-protein interaction analysis shed light on the network of interactions involving MSUD-related proteins. The Iranome database uncovered a potential pathogenic variant (c.554C>T) in the Persian population. This research contributes to a better understanding of MSUD genetics in the Iranian population and outlines potential avenues for further clinical investigations. The findings have implications for genetic testing, prognosis, and genetic counseling in affected families.

## INTRODUCTION

Maple syrup urine disease (MSUD), is an inherited condition that is distinguished by a lack of the branched-chain alpha-keto acid dehydrogenase enzyme complex, which is necessary for metabolizing the three branched-chain amino acids (leucine, isoleucine, and valine) in the body [1]. MSUD occurs in approximately 1 in 86,800 to 185,000 live birth [2, 3]. MSUD results in a distinct sweet odor in affected infants' urine. Symptoms of the disease include abnormal movements, delayed development, vomiting, lethargy, and poor feeding, Failure to treat the disease can result in seizures, coma, and death. MSUD can be classified as classic (severe), intermediate, or intermittent based on the severity of the condition. The classic type being the severe and most common form that appears shortly after birth. Newborns with classic MSUD do not initially show any symptoms, but if left untreated, the disease follows a predictable progression. Supplementary Table S1 shows the timeline of clinical information of a patient with classical MUSD over time [4]. 

Branched-chain ketoacid dehydrogenase (BCKAD) is a key enzyme involved in the catabolism of branched-chain amino acids (BCAAs) in various tissues, including kidney, liver, skeletal muscle, and brain. The BCKAD complex, comprising the E1, E2, and E3 catalytic subunits, resides in the inner mitochondrial membrane [4]. In MSUD, mutations in the genes encoding the BCKAD complex result in partial or complete dysfunction of the enzyme, thereby causing the build-up of BCAAs and their harmful byproducts in both the blood and tissues. The resulting metabolic imbalance can lead to a range of neurological symptoms and complications. Understanding the role of the BCKAD complex in the catabolism of BCAAs and its dysfunction in MSUD is crucial for understanding the pathophysiology of the disease [1, 3, 5, 6]. 

There are four specific gene variants that can be identified through molecular testing for MSUD: The *BCKDHA* gene is responsible for encoding the E1-alpha subunit of the BCKAD enzyme complex, which is associated with MSUD Type 1A. Likewise, the *BCKDHB* gene encodes the E1-beta subunit of the BCKAD enzyme complex, linked to MSUD Type 1B. Additionally, the *DBT* gene is accountable for producing the E2 subunit of the BCKAD enzyme complex, which is correlated with MSUD Type 2. Additionally, mutations in the *DLD* gene, which encodes a member of the class-I pyridine nucleotide-disulfide oxidoreductase family, have been found in individuals with E3-deficient MSUD and lipoamide dehydrogenase deficiency. The *PPM1K *gene was recently recognized as the fifth gene associated with MSUD. This gene is responsible for encoding a specific member of the PPM family of protein phosphatases that require the presence of Mn2+ and Mg2+ ions for their activity, which is associated with MSUD, Mild Variant and Intermediate MSUD [7]. Genetic testing provides valuable information for understanding the prognosis and genetic counseling of affected families [1]. In Iran, a comprehensive newborn screening initiative for MSUD and other inborn errors of metabolism (IEM) is absent, with the exception of phenylketonuria (PKU). Consequently, there is a scarcity of published information regarding the prevalence of the majority of IEMs in the nation [8]. MSUD follows an autosomal recessive inheritance pattern, and populations with a high prevalence of consanguineous marriages, like Iran, are anticipated to exhibit a higher incidence of IEMs [9]. In Iran, the rate of consanguineous marriages is reported to be 38.6% [10]. 

Iran is a country with a unique geographical location and a rich history of internal and external migrations, which has resulted in a diverse population consisting of various ethnicities [11, 12]. Despite this wide spectrum of diversity, there have been limited genetic studies conducted in Iran [10]. To fill the existing research gap, we conducted an in silico analysis focusing on the variation spectrum of the MSUD genes in a cohort of Iranian patients. The analysis involved a comprehensive examination of the reported variants identified through various molecular techniques such as PCR sequencing, PCR-RFLP, sanger sequencing, and VNTR allele analysis in Iranian patients with MSUD. Our objective was to investigate the pathogenic nature of these variants. Additionally, we explored the three-dimensional structure of the genes’ product, comparing the wild type with the mutant types associated with the identified variants. Through these analyses, we aimed to elucidate the clinical implications of the MSUD mutations. And we have outlined potential future directions for further research in this field [8].

## MATERIALS AND METHODS

**Data collection: **In order to gather a range of mutations within the among Iranian patients, a thorough systematic search was performed through various search engines. The search utilized specific keywords including "Iran", "BCKDHA", "BCKDHB", "DBT", "DLD", "PPM1K" and "MSUD" in both English and Persian, with a subsequent filtering of data to exclude reports lacking genetic testing, non-Iranian patients, and duplicated studies.

**Prediction of protein stability and variants pathogenicity: **The bioinformatics tools was employed to perform the prediction of protein stability and pathogenicity of genetic variations in Iranian individuals diagnosed with MSUD. These tools included the following: 1) Franklin, an online searchable framework specifically designed for interpreting genetic variants detected in clinical studies (https://franklin.genoox.com/clinical-db/), 2) CADD, a predictor of deleteri-ousness for both single nucleotide variants (SNV) and insertion/deletion (Indels) (https:// cadd.gs.washington.edu/), 3) Polyphen-2, utilized to anticipation of the consequences of missense mutations, on both the structure and function of proteins (http://genetics.bwh.harvard. edu/pph2/), 4) PANTHER, a protein Analyzer through evolutionary Relationships) (http:// pantherdb.org), 5) SIFT, which, based on sequence homology, anticipate the functional impact of amino acid substitutions (https://sift.bii.a-star.edu.sg/), 6) Mutation Taster, a free online tool used to assess DNA sequence variations for their potential to cause disease. (http://www. mutationtaster.org), 7) Fathmm, a functional consequences predictor of both coding and non-coding variants (http://fathmm.biocompute.org.uk/), 8) MuPro, a tool designed to predict the impacts of missense variations on protein solubility, flexibility, and stability (https://mupro. proteomics.ics.uci.edu/) and 9) iMutant 2, a software application designed to foresee alterations in protein stability stemming from point mutations (https://sls.cup.edu.in/ imutant2/).

In brief, the in silico analysis tools followed a defined criterion for prediction methodology. Variants that were identified as "deleterious/pathogenic" by all pathogenicity computational tools and indicated as "Decrease" by both protein stability in silico tools, MuPro and I-Mutant, were considered "high-risk pathogenic variants." These selected variants were then chosen for additional investigation.

**Amino acid conservation analysis: **In order to assess the conservation of amino acids within the high-risk pathogenic variants across different species, a conservation analysis was conducted using the Clustal Omega tool and the ConSurf web server. The protein's amino acid sequence from humans and other species was obtained from the HomoloGene sub-database of NCBI and submitted to Clustal Omega for multiple sequence alignment. The aligned sequences included those from *Danio rerio*, *Xenopus tropicalis*, *Gallus gallus*, *Bos taurus*, *Canis lupus familiaris*, *Macaca fascicularis*, *Pan troglodytes*, *Mus musculus*, and *Rattus norvegicus*. The ConSurf web server was utilized to predict the evolutionary patterns of amino and nucleic acids and to identify structural and functional areas based on conservation scores ranging from 1 to 9. Specifically, amino acid residues are posited to serve functional roles when they display a high level of conservation coupled with exposure, whereas structural roles are conferred upon residues that exhibit a high level of conservation in tandem with burial. 

**Secondary and tertiary structure prediction: **The utilization of PSIPRED (http://bioinf. cs.ucl.ac.uk/psipred/) has been extended to the authentication of the secondary structure of high-risk pathogenic variants. The PSIPRED server is established on distinct matrices that have been created via PSI-BLAST. The protein sequence in FASTA format was employed as input, and the output generated was in the form of secondary structures of the protein. This tool is commonly employed and involves the utilization of two artificial neural networks. The first network is utilized to forecast the likelihood of a particular amino acid being in helix, strand, or coil elements, while the second network applies these probabilities to forecast the overall secondary structure. In our study, we utilized the I-TASSER server (https://zhanglab.ccmb. med.umich.edu/I-TASSER/) to forecast the 3D configuration of both the wild and mutant types of The above proteins. This server is able to retrieve similar structures of a particular protein from the Protein Data Bank (PDB) and subsequently assemble them to form a 3D model. The predicted model is then subjected to molecular simulations and modeling techniques to fine-tune its energy and stability. Then, the ultimate outcome obtained from I-TASSER is displayed via the utilization of UCSF Chimera (https://www.cgl.ucsf.edu/chimera/). 

**Protein–protein interactions: **The STRING (https://string-db.org) was utilized to investi-gate the interaction of BCKDHA, BCKDHB, DBT, DLD and PPM1K proteins with each other and with other proteins. This tool forecasts the top ten proteins that exhibit interactions with the query gene, based on gene fusion, co-expression, function, and experimental data. It also presents composite scores for each interacting protein, which range from 0 to 1, with 0 denoting the lowest interaction and 1 indicating the highest interaction. 

**Analyzing hydrophobicity changes: **The analysis of alterations in hydrophobicity is conducted through the utilization of PEPTIDE 2.0 (https://www.peptide2.com/N_peptide_ hyd rophobicity_hydrophilicity.php), a web-based platform, and ExPASy/ProtScale, which employs the Kyte & Doolittle amino acid scale (https://web.expasy.org/protscale/). The estimation of protein structure parameters related to hydrophobicity or hydrophilicity relies on the physical and chemical characteristics of amino acids, determined using ExPASy/ProtScale.

**Predicting putative pathogenic variants utilizing Iranome database: **In this study, we utilized the Iranome Database (http://www.iranome.ir/), to identify potentially harmful variations in MSUD genes. The Iranome database was created through the examination of whole exome sequencing information derived from 800 healthy individuals representing eight significant Iranian population groups, such as Persians, Kurds, Lurs, Baluchs, Azeris, Iranian Arabs, Turkmen, and Persian Gulf Islanders. The database contains over 1.5 million variants, with more than 300,000 of these variants being novel. To evaluate the pathogenicity, the researchers used six tools (including SIFT, Polyphen2, Mutation Taster, Mutation Assessor, FATHMM, and FATHMM MKL). For each variant, predictions are listed on the Iranome website. We specifically focused on missense alleles found in *BCKDHA*, *BCKDHB* and *DBT* genes within the database to predict putative pathogenic variants. For each variant, we determined pathogenicity predictions, number of heterozygotes, and the populations with the highest and lowest allele frequency. To identify the putative pathogenic mutation most likely to occur in the Iranian population, we established strict criteria, which included the exclusion of variants with less than 10 heterozygotes, less than 20 of CADD score, and those predicted as pathogenic in less than 3 out of 6 web server predictors.

## RESULTS

**Figure 1:**
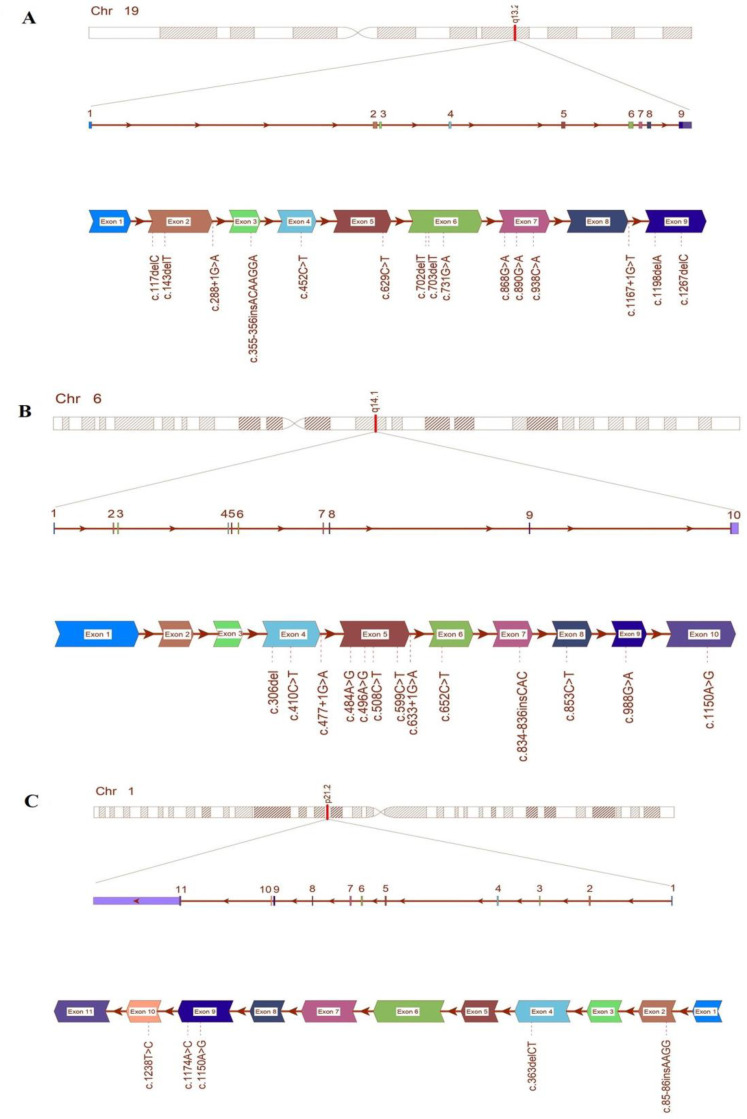
Graphical illustration of mutation spectrum of MSUD genes (*BCKDHA *(A), *BCKDHB *(B) and *DBT *(C)) in Iranian population.

The process of keyword research across multiple search engines yielded a comprehensive collection of 1179 manuscripts. However, through the use of inclusion criteria and the elimination of duplicate studies, it was discovered that only eleven of these manuscripts reported on homozygous variants in Iranian patients with MSUD. Following the filtering process, a total of 38 variants were identified, including 16 missense, 13 deletion, 4 insertion, 4 splicing, and 1nonsense mutations. The complete inventory of mutations in relation to the type of marriage, number of patients, and genetic test can be found in Supplementary Material 1. Furthermore, Figure 1 visually illustrates the spectrum of MSUD mutations in Iran.

In order to ascertain the pathogenicity and stability of all missense variants, a comprehensive analysis was conducted using nine distinct bioinformatic tools. The findings of this inquiry are meticulously documented in Table 1, which displays the outcomes of the pathogenicity and stability analysis of missense mutations. According to the table, the variants that were predicted to be high risk include 4 variants (R297H, G290R, G244E, A313D) in the *BCKDHA* gene and 6 variants (R170C, K166E, N162D, E330K, A137V, P218S) in the *BCKDHB* gene. Furthermore, Table 2 show cases the protein effects of nonsense, deletion, insertion, splice site and complex mutations, as reported in the literature.

**Table 1:** Prediction of pathogenicity and stability of missense mutations in Maple syrup urine disease (MSUD) genes in Iranian population

**Gene**	**Variant**	**Protein**	**ACMG**	**fathm**	**CADD**	**SIFT**	**POLY PHEN 2**	**Mutation taster**	**Mupro**	**I-Mutant 2.0**	**panther**	**References**
***BCKDHA***	c.629C>T	p.Ala210Val	LP	D (-5.61)	31	D	PD (1.000)	DC	Dec (DDG=-1.92)	Inc (DDG=0.27)	PD (0.74)	[13]
	c.890G>A	p.Arg297His	P	D (-3.81)	25	D	PD (0.999 )	DC	Dec (DDG=-0.62)	Dec (DDG=-0.61)	PD (0.95)	[14]
	c.868G>A	p.Gly290Arg	P	D (-4.53)	28.3	D	PD (1.000)	DC	Dec (DDG=-0.34)	Dec (DDG=-1.60)	PD (0.95)	[13]
	c.731G >A	p.Gly244Glu	LP	D (-8.70)	27.6	PD	D	DC	Dec (DDG=-0.63)	Dec (DDG=-0.44 )	PD (0.95)	[8]
	c.452C >T	p.Thr151Met	B	D (-3.88)	25.2	D	PD (0.937)	DC	Dec (DDG=-0.77)	Inc (DDG=0.20)	PD (0.57)	[15]
	c.938C >A	p.Ala313Asp	LP	D (-5.13)	29.7	D	PD (1.000)	DC	Dec (DDG= -0.46)	Dec (DDG=-1.68 )	PD (0.95)	[15]
***BCKDHB***	c.508G >T	p.Arg170Cys	P	D (-3.00)	_	D	PD (1.000)	DC	Dec (DDG=-0.76)	Dec (DDG=-1.01 )	PD (0.95)	[16]
	c.496A >G	p.Lys166Glu	LP	D (-2.94)	27.7	D	PD (1.00)	DC	Dec (DDG=-0.82)	Dec (DDG=-1.08 )	PD (0.89)	[15]
	c.484 A >G	p.Asn162Asp	LP	D (-2.96)	25.4	D	PD (1.000)	DC	Dec (DDG=-0.21)	Dec (DDG=-1.67)	PD (0.89)	[13]
	c.988G >A	p.Glu 330Lys	P	D (-3.22)	31	D	PD (1.000)	DC	Dec (DDG= -0.80)	Dec (DDG=-0.96 )	PD (0.95)	[15]
	c.410C>T	p.Ala137Val	P	D (-4.28)	25	D	PD (0.999 )	DC	Dec (DDG=-0.62)	Dec (DDG=-1.09)	PD (0.95)	[14]
	c.652C>T	p.Pro218Ser	VUS	D (-3.03)	27.5	D	PD (1.000)	DC	Dec (DDG=-1.01)	Dec (DDG=-1.36)	PD (0.95)	[14]
	c.599 C>T	p.Pro200L	LP	D (-2.67)	28.2	_	PD (0.917)	DC	Inc (DDG=0.41)	Dec (DDG=-0.11 )	PD (0.89)	[13]
***DBT***	c.1150A>G	p.Ser384Gly	B	_	21.7	D	B (0.083)	P	Dec (DDG=-1.20)	Dec (DDG=-0.77)	_	[14]
	c.1238 T >C	p.Ile413Thr	VUS	T (0.34)	25.0	D	PD (1.000)	DC	Dec (DDG=-1.42)	Dec (DDG=-2.14)	_	[13]
	c.1174 A >C	p.Thr392Pro	P	T (0.87)	16.26	D	D	DC	Dec (DDG=-1.38)	Dec (DDG=-1.62 )	_	[17]

**Abbreviations:** P, Pathogenic; LP, Likely Pathogenic; B, Benign; VUS, variants of unknown significance; D, Damaging; PD, Probably damaging ; T, Tolerated; DC, Disease causing; P, Polymorphism; Dec, Decrased; Inc, Incersed.

**Table 2:** Protein effects of splice site, deletion, duplication and complex mutations in MSUD genes in Iranian MSUD patients

**Gene**	**Variant**	**Reported Protein Effect**	**ACMG**	**References**
***BCKDHA***	c.117delC	p.R40Gfs*23	Pathogenic	[18]
	c.1267_1267delC	Q423fs	Likely Pathogenic	[15]
	c.355_356 ins 7 nt ACAAGGA	p.D335D fs	Likely Pathogenic	[13]
	c.703delT	p.Y235Tfs*95	Pathogenic	[13]
	c.702delT	p.Y235TfsX94	Likely Pathogenic	[8]
	c. 1167 + 1G>T	-	Likely Pathogenic	[8]
	c.(375 + 1 376-1) (884 + 1 885-1)	-	_-	[8]
	c.288 + 1G>A	-	Pathogenic	[8]
	c.1198delA	p.Lys400fs	Likely Pathogenic	[14]
	c. 143delT	p.L48RfsX14	Pathogenic	[8]
	c.143_143delT	L48fs	Pathogenic	[15]
***BCKDHB***	c.[633 + 1G > A]	-	Pathogenic	[16]
	c.833_834insCAC	p.Thr279dup	vus	[13]
	c. (274 + 1_275–1) _(343 + 1_344–1)del	-	_-	[16]
	c.[477 + 1 G > A]	-	_-	[19]
	c.(343 + 1_ 344–1)_(742 + 1_ 743–1)del	-	Pathogenic	[13]
	c.834_836dup CAC	p.Thr279dup	Pathogenic	[13]
	c.357delT	p.F120Lfs*110	Pathogenic	[13]
	c.853 C>T	p. R285X	Pathogenic	[8]
***DBT***	c.(433 + 1_ 434–1)_(939 + 1_ 940–1)del	-	Pathogenic	[13]
	c.[363delCT]	p.[Leu121Leu fs]	Pathogenic	[13]
	c.85_86ins AACG	p.V29Efs*21	Pathogenic	[13]

The Clustal Omega instrument was employed in the process of multiple sequence alignment of the BCKDHA and BCKDHB proteins of humans with other species. The outcomes of this study demonstrated that the amino acids identified in the areas of high-risk variations were exceedingly conserved in all other species, thereby pointing to the evolutionary and functional significance of the selected amino acids (Supplementary Fig. S1). The present study employed the utilization of the ConSurf tool to ascertain the degree of evolutionary conservation exhibited by amino acids in BCKDHA and BCKDHB proteins. The results indicate that most of 10 high-risk variations are highly conserved. The assessment reveals that four of the variants have obtained a conservation score of 9. The evaluation further indicates that two of the variants have been attributed with a score of 8, as three other variants have received a score of 7. Lastly, one of the variants has been assigned a score of 6. Three residues were predicted as structural and 3 others were classified as functional residues. (c).

The utilization of PSIPRED in forecasting and corroborating the secondary structure of the BCKDHA and BCKDHB proteins led to a combination of coil and alpha helix in the outcomes. It was observed that the major secondary structural motif was helix (50%), followed by coil (25%) and strand (25%) for BCKDHA. Also, the secondary structure of BCKDHB was predominantly helix (83.3%) with a smaller proportion of coil (16.6%), as depicted in Supplementary Fig. S3. To investigate the biophysical characteristics of high-risk variations, UCSF Chimera was employed for visualizing the alterations in amino acids. The analysis of these amino acid variations revealed no observable alterations in the polar contacts for the G290R, G244E, A137V, and P218S variants. In contrast, the polar contacts of the mutant residues in R297H, A313D, R170C, K166E, N162D, and E330K exhibited changes compared to the wild-type counterparts, as shown in Supplementary Figure S4.

**Figure 2:**
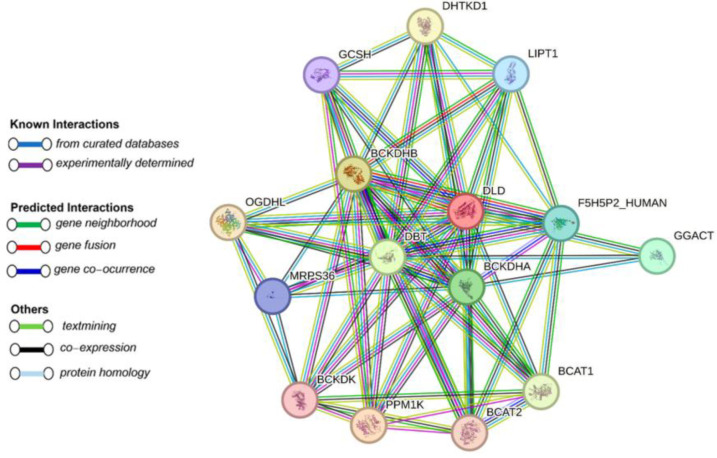
First shell (colored) and second shell (white) interactors of MSUD proteins predicted by protein–protein interaction analysis by STRING database.

The string tool was used to identify protein interactions. Initial results indicate that BCKDHA, BCKDHB, DBT, DLD and PPM1K proteins have the most interactions with each other and with proteins such as F5H5P2_HUMAN, BCAT2, OGDHL, GCSH, and DHTKD1 (Fig. 2). Proteins involved in causing MSUD participate in three common superpathways: Leucine, isoleucine and valine metabolism, Regulation of expression of SLITs and ROBO and Metabolic pathways. 

As demonstrated in Supplementary Table S2 it has been observed that the wild type residues at G290R, G244E and A313D amino acid substitutions exhibit greater hydrophobicity compared to the mutant residues. The reduction in hydrophobicity values has resulted in a decrease in the probability of hydrophobic interactions. It is noteworthy that such mutations have the potential to disturb the protein's structural integrity. The hydrophobicity adjustments, as calculated by ExPASy, were conspicuous in G290R, A313D, and P218S mutations, but insignificant in N162D, K166E, E330K and P218S mutations.

**Figure 3:**
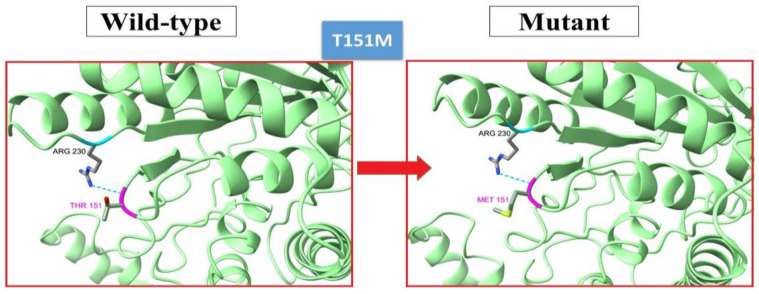
Tertiary structure of c.554C>T (p.Thr151 methionine) variants, which is found to be a putative pathogenic mutation in *BCKDHA* gene through the use of Iranome Genetic database.

Through an investigation into the Iranome, the *BCKDHA*, *BCKDHB* and *DBT* genes was scrutinized and all variations, including synonymous, missense, frameshift, 3'UTR, 5'UTR, splice site and intronic were identified. A total of 22 missense variations were uncovered, along with their corresponding highest and lowest allele frequencies within distinct populations in Iran, pathogenicity prediction, and number of hetrozygotes. These findings were summarized in Supplementary Table S3. By applying the established criteria as outlined in the methods, the c.554C>T variant was deemed a plausible pathogenic mutation within the *BCKDHA* gene. This variant was discovered to have the greatest allele frequency within the Iranian Persian population and the highest probability of occurring within MSUD patients resulting from marriage between two carriers. Fig. 3 displays the structural implications of this variant.

## DISCUSSION

MSUD is caused by mutations in four genes: *BCKDHA*, *BCKDHB*, *DBT*, and *DLD* which are involved in the metabolism of BCAAs. The most common mutations occur in *BCKDHA* and *BCKDHB*, reducing enzyme activity and causing toxic buildup of BCAAs. Mutations in the *DBT* gene also disrupt BCAA metabolism. Mutations occurring within the *DLD* gene result in intermittent branched-chain ketonuria, a specific form of MSUD. 

In the Iranian population, frequent mutations in *BCKDHA*, *BCKDHB*, and *DBT* genes have been observed, emphasizing their significance in MSUD. Understanding these mutations is crucial for genetic counseling, diagnosis, and tailored treatments in Iran. Ongoing research aims to further explore the range of mutations and their clinical implications in the Iranian context [20]. The catalytic domain in the BCKD enzyme is vital for its enzymatic activity and the breakdown of BCAAs. This domain facilitates the decarboxylation and oxidative decarboxy-lation reactions, converting BCAAs into acyl-CoA derivatives for energy production. Mutations in this domain can impair enzyme function, leading to disrupted BCAA metabolism and accumulation of toxic substances. most of the mutations occur in the catalytic domain of the BCKD enzyme. These mutations can disrupt its enzymatic activity, leading to impaired BCAA metabolism and the accumulation of toxic levels of BCAAs [21]. 

Through our analysis using Clustal Omega and Consurf, we found that all mutations in the BCKD enzyme occur in highly conserved regions across species. This indicates the functional importance of these regions in maintaining the enzyme's structure and function. The conservation of these regions throughout evolution underscores their significance in BCAA metabolism. The presence of mutations in these conserved regions highlights their potential impact on enzyme activity and the development of MSUD. This knowledge is valuable for understanding the molecular mechanisms underlying MSUD and can guide the development of targeted interventions to restore normal BCKD enzyme function [22]. 

In silico analysis utilizes computational tools to assess how genetic mutations affect protein function. This approach is valuable for comprehending disease mechanisms and identifying potential treatments. Compared to traditional laboratory-based methods, it offers a faster and more cost-effective means of analysis. In silico analysis also aids in the identification of previously unknown mutations. As a result, it is often the initial step in conducting functional studies. The objective of this research was to perform a computational analysis of the mutation patterns in the genes associated with MSUD disorder (*BCKDHA*, *BCKDHB*, and *DBT*) among Iranian patients diagnosed with MSUD. The study involved assessing documented genetic variations using PCR-sequencing techniques to determine the pathogenicity of various mutations. Additionally, we analyzed the protein structure and discussed the clinical implications, limitations, and potential future directions.

In this investigation, 58 patients were analyzed, and a total of 42 variants (42.8% located in *BCKDHA*, 45.2% in *BCKDHB*, and 11.9% in *DBT*) associated with MSUD disease were investigated. The results of this research indicate that in Iran, the *BCKDHB* gene is the primary locus for gene mutations in MSUD patients, with the *BCKDHA* and *DBT* genes subsequently identified as having a lower frequency of mutations. Moreover, 92.8% of these variants were identified in individuals from consanguineous marriages. PCR sequencing was the predominant method used, comprising 59.5% of cases, to examine gene mutations in patients with MSUD. By analyzing 20 missense variants using seven prediction software, it was found that all programs classified 10 mutations as deleterious. This implies a strong probability of these mutations being harmful. Consequently, the well-established MSUD mouse model can serve as a valuable resource for conducting experimental and functional studies to explore these mutations further and understand their potential involvement in disease development [23, 24].

Research has demonstrated the connection between protein function, structural alterations, and the emergence of new phenotypes. Gaining knowledge about the structural changes in protein dynamics is crucial for comprehending the molecular implications of mutations. Mutations and alterations in protein sequence generally impact the function, stability, and structure of proteins. Specifically, mutations in *BCKDHA*, *BCKDHB*, and *DBT* genes result in reduced activity and stability of the BCKD enzyme, while certain mutations also induce changes in its oligomeric state [22, 25]. 

The Iranome database (http://www.iranome.ir/) provided information on 22 missense variants in disease-related genes (*BCKDHA*, *BCKDHB*, and *DBT*), which were analyzed to determine their potential impact on protein function. Multiple functional prediction tools predicted some of these variants to be damaging or probably damaging, with varying frequencies across different populations. Among these variants, one specific variant (c.554C>T) met the criteria for being a potential pathogenic variant. This variant was found to have the highest allele frequency in the Persian population and the lowest frequency in the Lur population. Given its potential pathogenicity and high frequency in the Persian population, implementing carrier screening may serve as a viable approach to mitigate the occurrence of affected offspring within this particular group. It is important to note that MSUD is an autosomal recessive disorder, and consanguineous marriages increase the likelihood of children inheriting two copies of disease-causing variants in MSUD genes. Therefore, genetic counseling is crucial for affected families, emphasizing the need for informed decision-making.

Although in silico analysis is valuable in predicting the potential effects of genetic variations, functional studies are necessary to validate these predictions accurately. Precisely identifying the specific mutation in a patient is vital for clinicians to prescribe the most effective treatment pathway, facilitate timely and optimal therapies, and avoid unnecessary use of ineffective medications. Strip assays offer convenient and cost-effective solutions for detecting the most frequent mutations. Our study has contributed to this by creating a list of the most common mutations in *BCKDHA*, *BCKDHB*, and *DBT* genes. Additionally, considering the high frequency of c.554C>T in the Persian population, it would be a suitable candidate for inclusion in a strip assay kit. Designing such a test would be the most appropriate approach to efficiently identify gene mutations related to MSUD in the Iranian population in a timely manner.
