# Supplementary material for: A comprehensive in silico analysis of mutation spectrum of maple syrup urine disease (MSUD) genes in Iranian population
Source: Mol Biol Res Commun. 2024;13(4):235–46. doi: 10.22099/mbrc.2024.49847.1958 (PMC11416852; doi:10.22099/mbrc.2024.49847.1958)
Supplement: Supplementary Figures [file mbrc-13-235.s001.pdf]

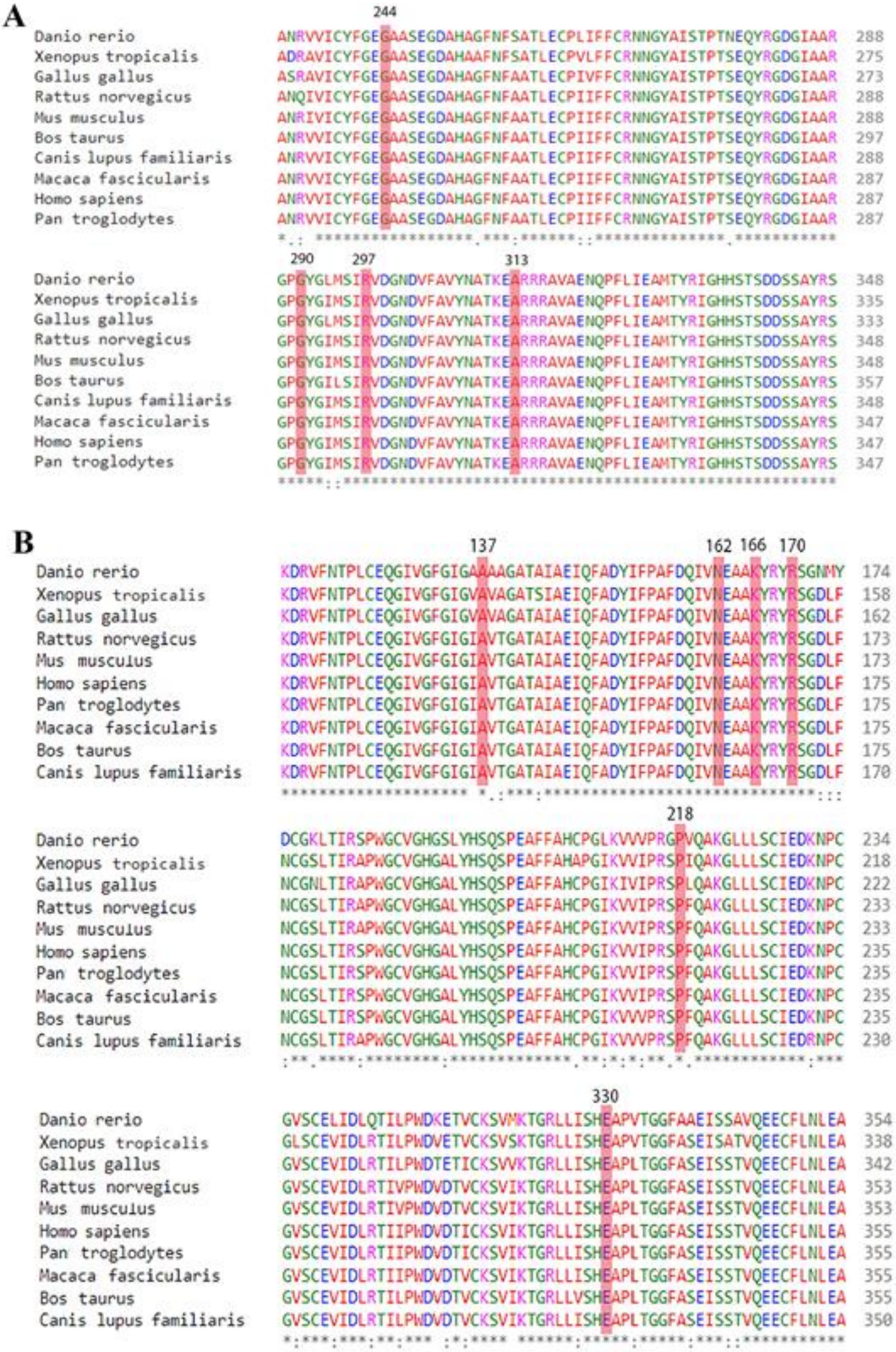

Figure S1: Amino acid conservational study of BCKDHA(A) and BCKDHB(B) proteins sequence using Clustal Omega across 10 different species. High-risk variants are marked in red.

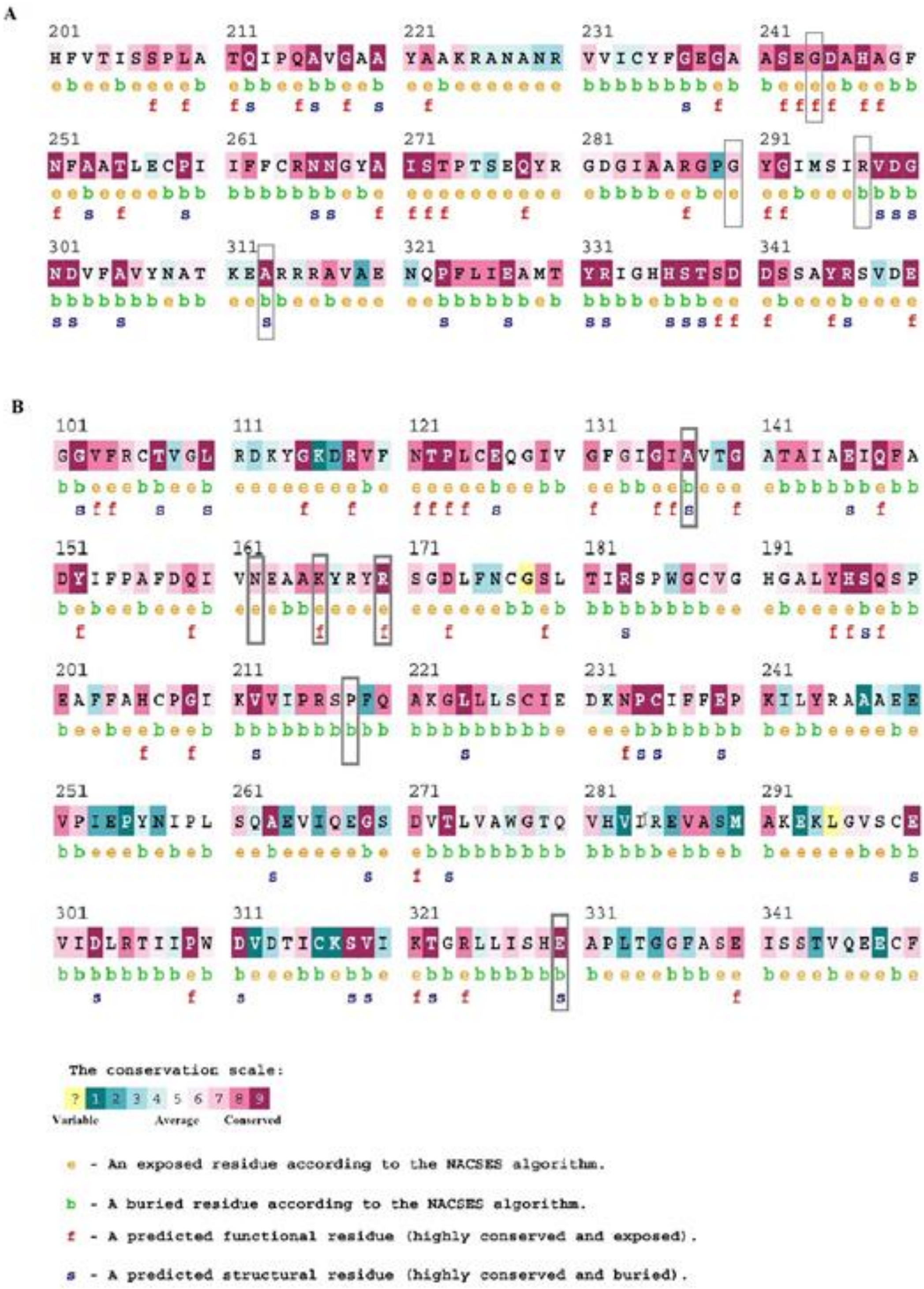

Figure S2: Evolutionary conservation profile of BCKDHA(A) and BCKDHB(B) proteins according to Consurf web server, showing by sequence. The gray boxes indicate the high-risk variants.

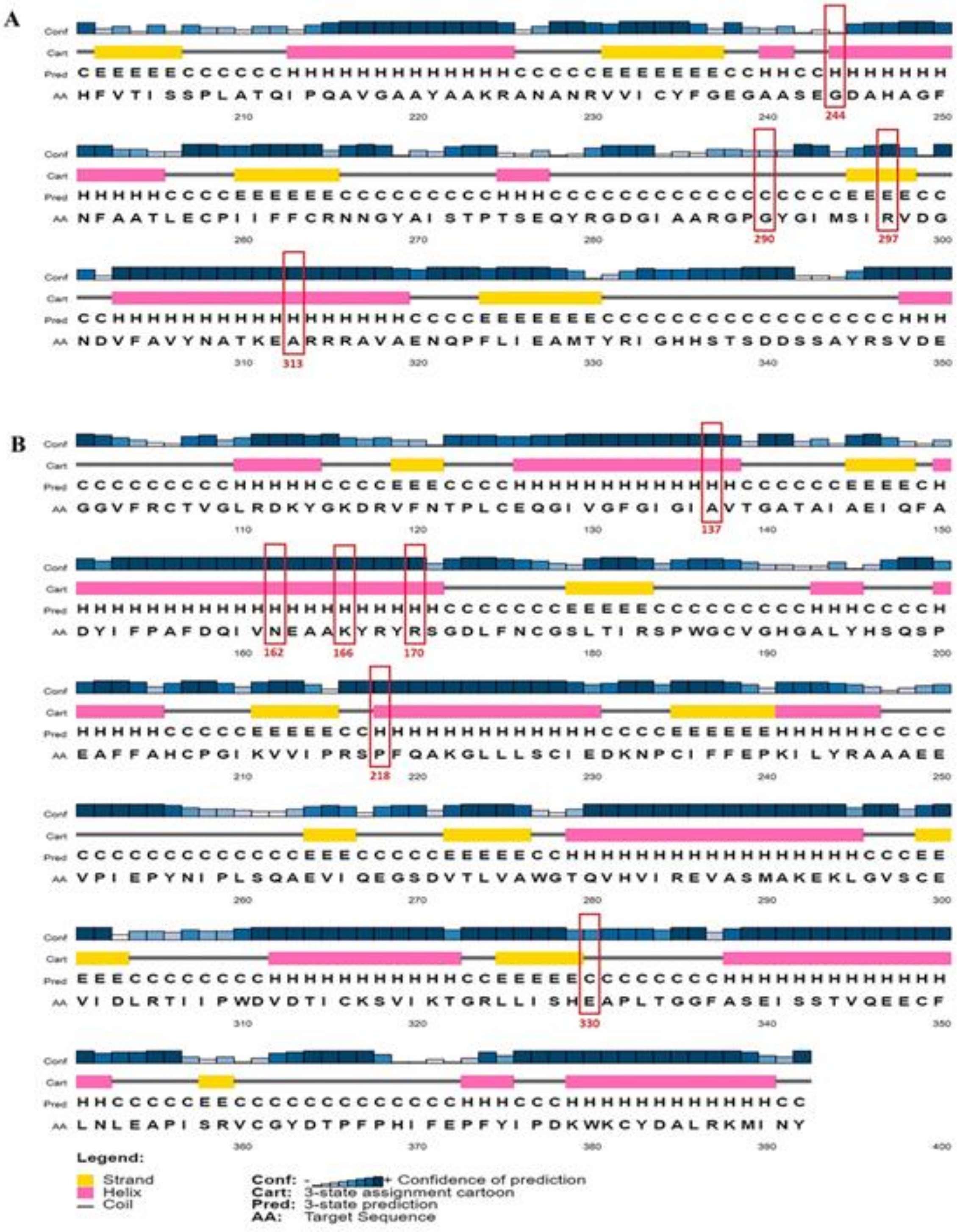

Figure S3: Secondary structure of BCKDHA(A) and BCKDHB(B) proteins created by PSIPRED web server.Red rectangles shows the exact location of deemed pathogenic missense variants in our cohort.

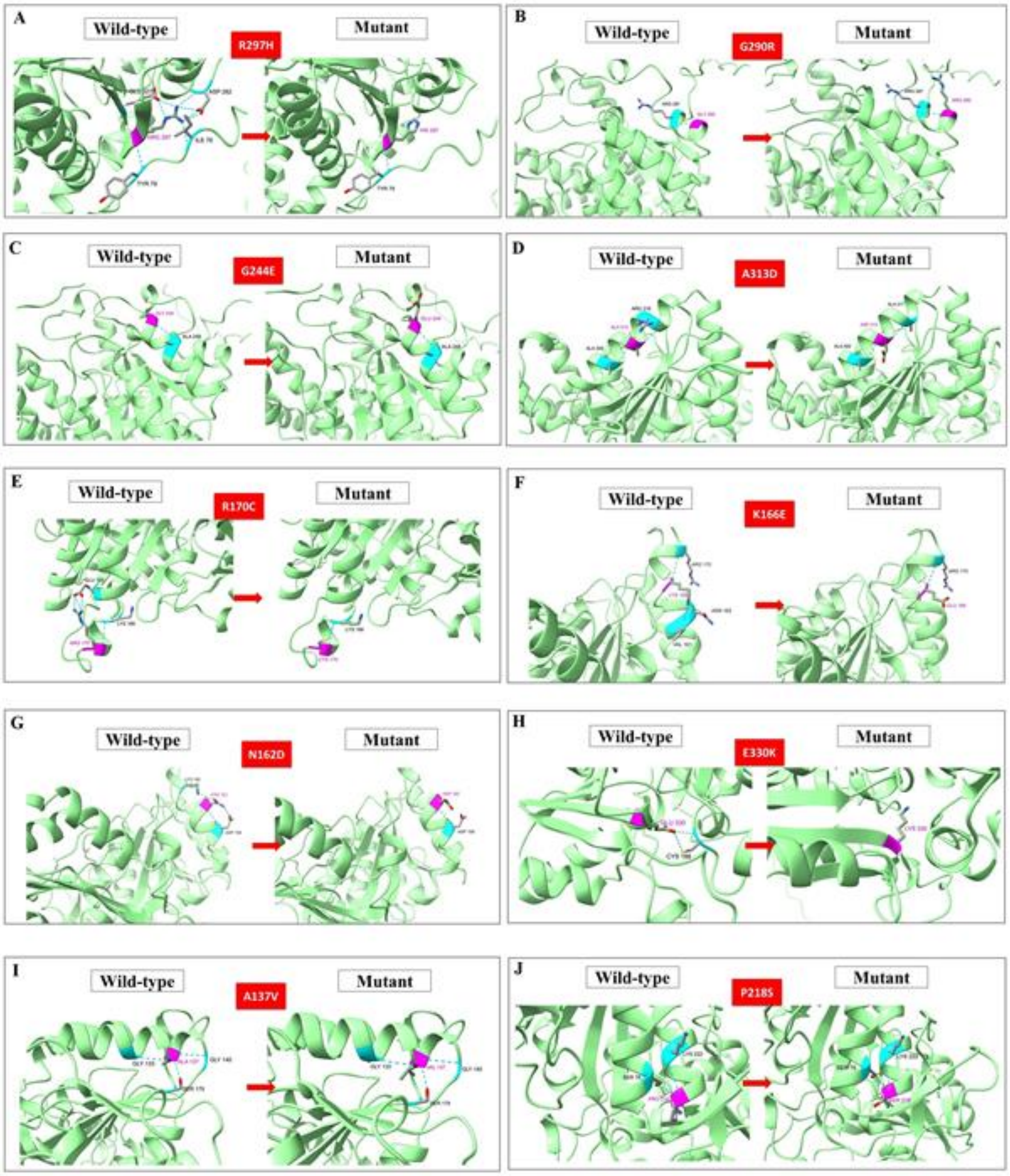

Figure S4: Effect of the high-risk variants in our cohort on 3D structure of BCKDHA(A-D) and BCKDHB(E-I) proteins.
