# Supplementary material for: A comprehensive in silico analysis of mutation spectrum of maple syrup urine disease (MSUD) genes in Iranian population
Source: Mol Biol Res Commun. 2024;13(4):235–46. doi: 10.22099/mbrc.2024.49847.1958 (PMC11416852; doi:10.22099/mbrc.2024.49847.1958)
Supplement: Supplementary Tables [file mbrc-13-235.s002.pdf]

Table S1: Clinical features of classical MSUD over time.

Time Period	Clinical Features	Underlying Pathophysiology	Consequences	Management Strategies
**12-24 hours**	Elevated concentrations of BCAAs (leucine, isoleucine, and valine) and alloisoleucine present in blood; Generalized disturbance of amino acid concentration ratios; Maple syrup odor detected in cerumen	Deficiency of branched-chain alpha-keto acid dehydrogenase enzyme complex; Buildup of BCAAs and their toxic byproducts in the blood	Generalized disturbance of amino acid metabolism; Potential for neurological damage if left untreated	Diagnosis via newborn screening; Initiate treatment with specialized formula; Restriction of dietary intake of BCAAs
**2-3 days**	Irritability, hypersomnolence, anorexia; Branched-chain alpha-ketoacids, acetoacetate, and beta-hydroxybutyrate present in urine	Continued buildup of BCAAs and their toxic byproducts; Metabolic acidosis due to accumulation of ketoacids	Progression of neurological symptoms; Increased risk of seizures and coma; Potential for renal dysfunction	Continued dietary restriction of BCAAs; Supplementation with thiamine, biotin, and other vitamins; IV glucose administration
**4-6 days**	Lethargy, apnea, opisthotonos, reflexive "fencing" or "bicycling" movements; Sweet maple syrup odor apparent in urine	Worsening of neurological symptoms due to accumulation of BCAAs in the brain	Increased risk of seizures, coma, and brain damage; Potential for respiratory failure	Initiate hemodialysis to rapidly reduce BCAA levels; Intensive supportive care; Consider liver transplantation in severe cases
**7-10 days**	Critical cerebral edema, coma, central respiratory failure	Severe brain damage due to prolonged metabolic derangement	High risk of mortality	Aggressive supportive care; Consider palliative care measures if prognosis is poor

Table S2: Evaluation of the hydrophobicity changes via the PEPTIDE 2.0 tool and ExPASy resource portal.

**gene**	**Amino acid change**	**PEPTIDE-2 prediction,Hydrophobicity index*(%)**	**Change of the nature of the amino acid**	**Hydrophobicity change in substituted position by ExPASy resource portal**
BCKDHA	p.R297H	not change	Hydrophilic to Hydrophilic	0.144
	p.G290R	Basic: 15.06/Neutral: 33.03	Neutral to Hydrophilic	-0.456
	p.G244E	Acidic: 11.46/Neutral: 33.03	Neutral to Hydrophilic	-0.344
	p.A313D	Hydrophobic: 40.45/Acidic: 11.46	Hydrophobic to Hydrophilic	-0.589
BCKDHB	p.R170C	Basic: 11.48/Neutral: 30.1	Hydrophilic to Hydrophobic	-0.778
	p.K166E	Acidic: 11.22/Neutral: 29.85	Hydrophilic to Hydrophilic	-0.044
	p.N162D	Acidic: 11.22/Neutral: 29.59	Hydrophilic to Hydrophilic	0
	p.E330K	Acidic: 10.71/Basic: 11.99	Hydrophilic to Hydrophilic	-0.045
	p.A137V	not change	Hydrophobic to Hydrophobic	0.266
	p.P218S	Hydrophobic: 47.19/Neutral: 30.1	Hydrophilic to Neutral	-0.089

Table S3: Putative pathogenic variants in MSUD genes identified through the use of Iranome genomic database.

	missense variants	Number of Heterozygotes	CADD Score	SIFT Pred	Polyphen2 HVAR Pred	MutationTaster Pred	MutationAssessor Pred	FATHMM Pred	FATHMM MKL Coding Pred	N of 6 Damaging Prediction	Population with the most Frequency of Allele	Population with the Least Frequency of Allele
BCKDHA	c.98C>T	1	16.91	Damaging	Benign	Tolerated	Predicted non-functional (neutral)	Damaging	Tolerated	2	Turkmen(0.005)	Persian Gulf Islander(0)
	c.113T>C	3	0.951	Tolerated	Benign	Tolerated	Predicted non-functional (neutral)	Damaging	Tolerated	1	Baloch(0.01)	Turkmen(0)
	c.218C>A	193	2.132	Tolerated	Benign	Tolerated	Predicted non-functional (neutral)	Damaging	Tolerated	1	Baloch(0.185)	Persian(0.115)
	c.554C>T	40	30	Damaging	Possibly damaging	Damaging	Predicted non-functional (low)	Damaging	Damaging	5	Persian(0.045)	Lur(0.005)
	c.1012T>C	0	8.084	Tolerated	Benign	Damaging	Predicted non-functional (neutral)	Damaging	Damaging	3	Arab(0)	Turkmen(0)
	c.1043G>A	1	35	Damaging	Probably damaging	Damaging	Predicted functional (medium)	Damaging	Damaging	1	Arab(0.005)	Turkmen(0)
	c.1172A>G	1	12.39	Tolerated	Benign	Damaging	Predicted non-functional (neutral)	Damaging	Damaging	3	Azeri(0.005)	Turkmen(0)
	c.1313A>G	2	8.991	Tolerated	Benign	Tolerated	Predicted non-functional (neutral)	Damaging	Tolerated	1	Turkmen(0.01)	Persian Gulf Islander (0)
	c.1349T>C	1	20.6	Damaging	Benign	Damaging	Predicted functional (medium)	Damaging	Damaging	5	Azeri(0.005)	Turkmen(0)
	c.1379C>T	1	25.1	Damaging	Benign	Damaging	Predicted non-functional (low)	Damaging	Damaging	4	Turkmen(0.005)	Persian Gulf Islander (0)
BCKDHB	c.13G>T	2	8.256	Tolerated	Benign	Tolerated	Predicted non-functional (neutral)	Damaging	Tolerated	1	Lur(0.0101)	Turkmen(0)
	c.115G>A	1	0.002	Tolerated	Benign	Tolerated	Predicted non-functional (neutral)	Damaging	Tolerated	1	Lur(0.005)	Turkmen(0)

	c.410C>T	1	32	Damaging	Probably damaging	Damaging	Predicted functional (high)	Damaging	Damaging	6	Arab(0.005)	Turkmen(0)
	c.854G>A	1	18.35	Tolerated	Benign	Damaging	Predicted non-functional (neutral)	Damaging	Damaging	3	Azeri(0.005)	Turkmen(0)
	c.946T>C	1	20.2	Tolerated	Possibly damaging	Damaging	Predicted non-functional (low)	Damaging	Damaging	4	Lur(0.005)	Turkmen(0)
DBT	c.1418A>G	1	15.75	Damaging	Benign	Damaging	Predicted non-functional (low)	Tolerated	Damaging	3	Lur(0.005)	Turkmen(0)
	c.1380G>A	1	23.7	Tolerated	Benign	Damaging	Predicted non-functional (low)	Tolerated	Damaging	2	Azeri(0.005)	Turkmen(0)
	c.1150A>G	120	1.112	Tolerated	Benign	Tolerated	_	Tolerated	Tolerated	0	Baloch(0.95)	Lur(0.89)
	c.724T>C	10	1.704	Tolerated	Benign	Tolerated	Predicted non-functional (neutral)	Tolerated	Tolerated	0	Lur(0.02)	Turkmen(0)
	c.715A>G	2	0.004	Tolerated	Benign	Tolerated	Predicted non-functional (neutral)	Tolerated	Tolerated	0	Arab(0.005)	Turkmen(0)
	c.535C>T	2	35	Damaging	Probably damaging	Damaging	Predicted functional (high)	Tolerated	Damaging	5	Persian Gulf Islander(0.02)	Turkmen(0)
	c.107A>T	2	11.36	Tolerated	Benign	Tolerated	Predicted non-functional (neutral)	Tolerated	Damaging	1	Kurd(0.005)	Turkmen(0)
